# The effectiveness of self help technologies for emotional problems in adolescents: a systematic review

**DOI:** 10.1186/1753-2000-2-20

**Published:** 2008-07-23

**Authors:** Muna Ahmead, Peter Bower

**Affiliations:** 1 School of Public Health, Al-Quds University, Jerusalem, Israel; 2 NPCRDC, 5th Floor, Williamson Building, University of Manchester, M13 9PL, UK

## Abstract

**Background:**

Adolescence is a transition period that involves physiological, psychological, and social changes. Emotional problems such as symptoms of anxiety and depression may develop due to these changes. Although many of these problems may not meet diagnostic thresholds, they may develop into more severe disorders and may impact on functioning. However, there are barriers that may make it difficult for adolescents to receive help from health professionals for such problems, one of which is the limited availability of formal psychological therapy. One way of increasing access to help for such problems is through self help technology (i.e. delivery of psychological help through information technology or paper based formats). Although there is a significant evidence base concerning self help in adults, the evidence base is much weaker in adolescents. This study aims to examine the effectiveness of self help technology for the treatment of emotional problems in adolescents by conducting a systematic review of randomized and quasi-experimental evidence.

**Methods:**

Five major electronic databases were searched: Medline, PsycInfo, Embase, Cochrane Controlled Trials Register and CINAHL. In addition, nine journals were handsearched and the reference lists of all studies were examined for any additional studies. Fourteen studies were identified. Effect sizes were calculated across 3 outcome measures: attitude towards self (e.g. self esteem); social cognition (e.g. self efficacy); and emotional symptoms (i.e. depression and anxiety symptoms).

**Results:**

Meta analysis showed small, non-significant effect size for attitude towards self (ES = -0.14, 95% CI = -0.72 to 0.43), a medium, non-significant effect size for social cognition (ES = -0.49, 95% CI = -1.23 to 0.25) and a medium, non-significant effect size for emotional symptoms (ES = -0.47, 95% CI = -1.00 to 0.07). However, these findings must be considered preliminary, because of the small number of studies, their heterogeneity, and the relatively poor quality of the studies.

**Conclusion:**

At present, the adoption of self help technology for adolescents with emotional problems in routine clinical practice cannot be recommended. There is a need to conduct high quality randomised trials in clearly defined populations to further develop the evidence base before implementation.

## Background

Adolescence is considered a challenging stage of life. It is a transition period from childhood to adulthood that involves physiological changes, developments in cognition and emotion, changes in social roles with peers and the opposite sex, and considerations of school and career. It involves the development of identity, independence from family and adaptation to peer groups [1]. If children and adolescents struggle to cope with these changes, they may develop emotional disorders, such as anxiety, depression and obsessions [2,3].

Depression covers a range of personal moods from a mild case of the 'blues' to clinical conditions that are characterized by severe symptoms and functional impairments [4]. Data collected for The Youth Risk Behavior Surveillance System found that in the United States, during the 12 months preceding the survey, 28.5% of students had felt so sad or hopeless almost every day for more than 2 weeks in a row that they stopped doing some usual activities [5]. Prevalence of depression reaching diagnostic thresholds is estimated at around 0.4–8% in adolescents over 12 months [6,7].

Similarly, anxiety problems range from presence of symptoms to clinical conditions such as separation anxiety, social phobia, generalized anxiety disorder, obsessive compulsive disorder, panic disorder and phobias [4,8]. Prevalence rates for having at least one childhood anxiety disorder vary, with 12 month estimates in the United States and internationally from 8.6% to 20.9% [9]. Adolescents with elevated but subsyndromal levels of anxiety symptoms report significant levels of functional impairment [10,11].

Depression and anxiety in children and adolescents have a large number of potential consequences including academic failure, poor peer relationships, behavioural problems, conflict with parents, substance abuse [12] recurrent anxiety or depressive disorders [13] and suicide attempts [14].

There are a number of studies that show the effectiveness of cognitive behaviour therapy for adolescents with clinical depression [15-19], although combination treatment with medication may be optimal [20]. CBT also has an important role in the management of anxiety [21]. However access to psychological therapy is limited, and is appropriately targeted at those with more severe disorders. This raises the importance of alternative solutions, especially for those who have early symptoms of anxiety, depression or emotional distress (such as poor peer relations, low self-esteem, withdrawal or behavioural problems) but do not reach formal diagnostic thresholds [22].

One option is the use of self help treatments. In mental health, self help is seen as 'the manualization of evidence based treatment' [23]. This involves taking aspects of proven treatments and providing them through technology, such as information technology and written paper-based formats.

Current clinical guidelines in the United Kingdom suggest guided self help technology could be a useful treatment for some emotional problems in adolescents [24]. A number of randomized controlled trials and systematic reviews have indicated that self help technologies are helpful for adult patients [25-29]. However, the evidence base is far weaker in relation to adolescents.

This study aimed to determine the effectiveness of self help technology for the treatment of emotional problems in adolescents using systematic review methods.

## Methods

### Inclusion and exclusion criteria

#### Study design

Randomized controlled trials (RCT) and quasi-experimental studies which used a control group (e.g. usual care, placebo controls, waiting list controls, or no treatment controls) were eligible for the review.

#### Populations

Adolescence was defined as age between 12–25 years to cover the wide variation in the definition of adolescence in the literature [30-34].

#### Disorders

Emotional symptoms including depression [35] and anxiety [35,36] were included. These could be symptoms or disorders severe enough to reach diagnostic thresholds. Two other outcomes were also included: attitude towards self, including self concept [37] and self esteem [38]; and social cognition, such as self efficacy and locus of control [39]. Both of these concepts may be important causes or consequences of emotional problems [40-45].

#### Interventions

Self help materials were interventions delivered through information technology (e.g. web-based or stand alone computer programs); paper-based delivery (i.e. bibliotherapy); audiotapes or videotapes. For inclusion, the materials had to be used by the participants with no or minimal individual contact with a health professional or researcher.

### Search strategy

**Figure 1:**
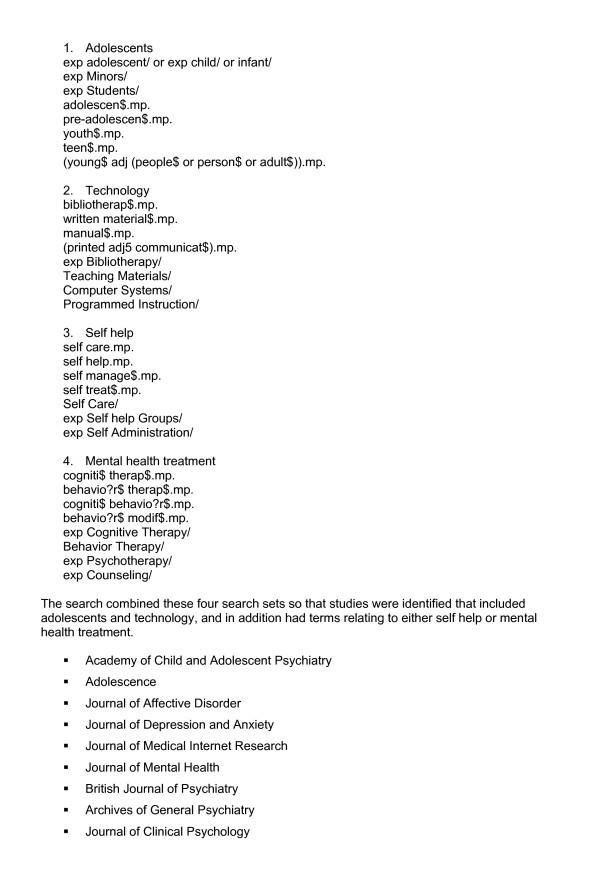
**Example search strategy (MEDLINE) and list of handsearched journals**. The search was structured to search for studies with four key characteristics: adolescents, technology, self help and mental health treatment. Each search used a combination of free text and subject headings. Examples are given below, and the entire search strategy is available from the authors. The search combined these four search sets so that studies were identified that included adolescents and technology, and in addition had terms relating to either self help or mental health treatment. The listed journals were hand searched from first issue 1995 to March-May 2005.

The strategy involved searching five major electronic databases: Medline (1966 onwards), PsycInfo (1967 onwards), Embase (1980 onwards), CINAHL (1982 onwards) and the Cochrane Controlled Trials Register. These databases were searched initially in late 2004 and early 2005, and then updated in April 2006. Specific searches were developed for each database to maximise the effectiveness of each search. The search terms were grouped into those concerning the adolescent population (e.g. Adolescent, Minors), those related to the technology (e.g. Bibliotherapy, Internet) and those related to the intervention, either self help (e.g. Self Care, Self Administration) or mental health treatment (e.g. Cognitive Therapy, Counselling). Further details are provided in Figure 1. The search strategy was chosen to maximise sensitivity at some loss of specificity. For example, study design terms were not included in the search strategy because some quasi-experimental studies may not be correctly indexed. Similarly, terms related to problems or disorders such as depression and anxiety were also excluded from the search strategy, and a decision was made to identify all possible studies on the basis of population, technology and intervention only. Relevant studies were then included or excluded on the basis of study design or disorders after reading titles and abstracts.

Nine journals which were identified on the basis of scoping searches as publishing papers on self help and related topics were hand searched from 1995 to March-May 2005 (see Figure 1). The reference lists of all studies were examined for any additional studies, and Google Scholar was searched regularly for any relevant studies from 2004 to 2006. Only studies reported in the English language were included because of the lack of a budget for translation.

The first author checked the titles and abstracts of all studies identified by the searches. Any studies that were judged potentially eligible were set aside for discussion by both authors before a final decision about inclusion was made.

### Data extraction

A data extraction sheet was developed and data were extracted by two independent reviewers, with disagreements resolved by discussion or contact with the authors. The data extraction sheet included the following groups of variables: study data variables, methodological variables, population variables, and intervention and outcome variables.

### Quality assessment

In this review, quality assessment including internal validity (i.e. study design, conduct and analysis) and external validity (i.e. recruitment of the population) were assessed using the Quality Rating Scale (QRS) rated by two researchers independently [46]. The QRS consists of 23 items. Each item is scored 0, 1, or 2 and the total score ranges from 0–46. In this study, two items were excluded because they were not applicable ('blinding of participants' and 'treatment side effects') [47]. The reliability of the QRS ratings was assessed by measuring inter-observer agreement using the kappa statistic [48].

### Data analysis: Computation of effect size

The meta analysis used the standardized mean difference estimate of effect size. Effect size for each study was calculated by subtracting the control group mean from the experimental group mean, and dividing by the pooled standard deviation (SD) [49]. Where relevant statistics (e.g. standard deviation) were not available, effect size was calculated from other indices using published methods [49].

The main comparison was between self help technology versus no treatment or delayed treatment control group. Only one outcome measure was selected from each study according to its relevance to the 3 major outcomes (emotional symptoms, attitude towards self and social cognition).

Both fixed effects and random effects model were used in calculating the overall effect size using the *metan *routine within Stata. The former assume that the effect of treatment is the same across studies and that any difference between their results is due to sampling error, while the latter assume that the effect of treatment is not the same across studies and that variation in treatment effect between studies occurs as a result of factors other than sampling error [49,50]. Both models give the same results when there is no significant heterogeneity. When marked heterogeneity is present, the random effects model produces wider confidence intervals and may produce a different estimate of effect [51,52]. Heterogeneity was assessed using the I^2 ^statistic [53].

## Results

**Figure 2:**
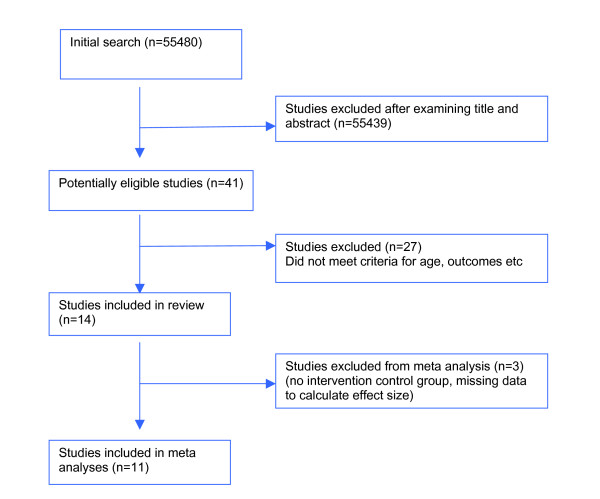
Review search flowchart.

A total of 55,480 studies were identified (figure 2). As noted above, the searches were designed to maximise sensitivity over specificity, and many studies identified in the initial search were excluded when scanning the abstracts because they failed to meet the criteria for study design and disorder. Fourteen studies eventually met the inclusion criteria. The review found six studies (listed in table 1) involving anxiety (including test and dating anxiety), 2 studies involving depression, and six involving other problems related to anxiety and depression (e.g. self esteem, problem solving skills). Thirteen studies were conducted in the USA and one in Australia.

**Table 1:** Characteristics of the included studies

**Study**	**Study design**	**Target population and outcomes**	**N**	**Age**	**Sex**	**Follow up (weeks)**	**Attrition**	**QRS score**
Ackerson [54]	RCT	Adolescents with elevated depression symptoms (10+ on the Child Depression Inventory and 10+ on the Hamilton Rating Scale for Depression)	22	Mean 15 years and 11 months	M: 36%	4 weeks follow up	26.6%	21
Allen [65]	Quasi-experiment	Students with test anxiety	84	17–21	M: 25%	7 weeks	24%	14
Buglione [55]	Quasi-experiment	Students with test anxiety (scoring above the 60th percentile on the Test Anxiety Inventory)	50	18–22	M: 58%	Post treatment, but minimum of 6 weeks	28%	17
Denny [62]	Quasi-experiment	Students with spider fear (scoring in the upper 20th percentile of a spider fear inventory and failing a behavioural avoidance test)	70	18–21	M: 10%	Not clear (week following last therapy session)	Not clear	13
Grossman [58]	RCT	Male students with dating anxiety	50	18–21	M: 100%	4 weeks and 8 months follow up	8%	18
Lenkowsky [60]	Quasi-experiment	Self concept in students with learning disability and emotional handicap	96	12–14	M: 83%	Not clear (post treatment)	Not clear	11
O'Kearney [56]	Quasi-experiment	Students with depression symptoms	78	15–16	M: 100%	8–10 weeks and 16 weeks follow up	41%	23
Ramsey [63]	Quasi-experiment	Stress management training in students	132	18 – 23+	M: 47%	4 weeks and 4 weeks follow up	12%	16
Register [57]	RCT	Students with test anxiety (meeting Test Anxiety Inventory criterion cut off score of 50)	121	Mean 18.6	M: 31%	Treatment completion, 4 week follow up	8%	16
Robinson [66]	RCT	Health behaviour change in graduate and undergraduate students	952	18–25	Not clear	16 weeks	23%	16
Salt [59]	Quasi-experiment	Self esteem and locus of control in high school students	121	14–17	M: 46%	2 weeks	13%	12
Sandor [37]	RCT	Problem solving in adolescents living with single parent mothers who had been separated or divorced from fathers for approximately 6–48 months	100	13 – 17	M: 37%	4 weeks post intervention and 4 weeks follow up	3%	24
Sheridan [61]	RCT	Prevention of problems in youth of changing families	48	13–15	M: 48%	Not clear	Not clear	13
Walker [64]	Quasi-experiment	Competence building in adolescents in church youth and schools	139	13–18	M: 40%	2 months	22%	15

### Characteristics of the participants

Students were the target populations in most studies (n = 12). Studies used volunteer participants who were recruited using advertisements, letters, and contact with school personnel or through course entry. Only two studies reported the inclusion of clinical participants [54,55], and only two studies reported the number of the eligible participants who did not participate in the study to judge their representativeness [56,57]. Most of the studies included both genders, but with more females than males, although two studies had males only [56,58].

### Description of the interventions

Details of the interventions are shown in Table 2 [see Additional file 1]. Four studies used computer interventions, 8 studies used bibliotherapy and 2 studies used videotaped interventions. Seven studies delivered interventions through specific group sessions (duration between 30 to 60 minutes per session) in which the participants worked alone on self help interventions or with minimal instructions from the therapists or researchers [55,56,59-63]. In the other seven studies, the participants read the materials alone at home and they either had at least one telephone call per week during the intervention period [37,54,64,65], were sent a newsletter [66] or received two letters and one telephone call as a reminder [58]. One study had no contact [57].

### Quality assessment of included studies

Six studies were RCTs [37,54,58,61,64,66] and eight studies were quasi-experiments [55,56,59,62-65]. Inter-observer agreement on quality ratings assessed using the kappa statistic was 'substantial' (0.77). The mean quality score of all included studies was low (16.4 out of 42). Table 1 shows the overall quality score of each study.

The low quality score of the studies included in this meta analysis occurred as a result of a wide range of methodological weaknesses. For example, studies generally had small sample sizes (less than 50 participants per group); none reported concealment of allocation; few reported power calculations; and all had follow up periods of less than 6 months.

### Effect size estimation

Meta analysis was used to determine the overall effectiveness of self help technology. The analyses indicated significant statistical heterogeneity for attitude towards self (chi-squared = 23.39, d.f. = 4, p = 0.000, I^2^= 82.9%), emotional symptoms (chi-squared = 25.38, d.f. = 6, p = 0.000, I^2^= 76.4%) and social cognition (chi-squared = 16.63, d.f. = 2, p = 0.000, I^2 ^= 88.0%). Therefore, analyses were conducted using random effects models.

**Figure 3:**
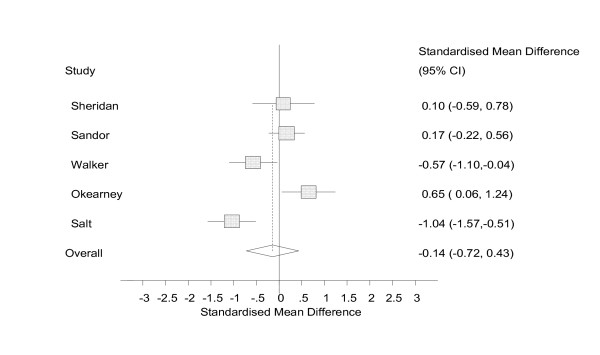
Analysis of attitude towards self, random effects.

**Figure 4:**
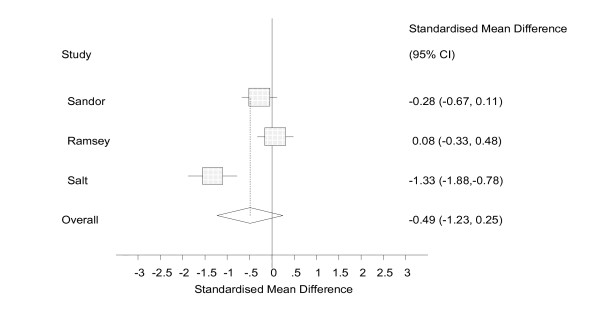
Analysis of social cognition, random effects.

**Figure 5:**
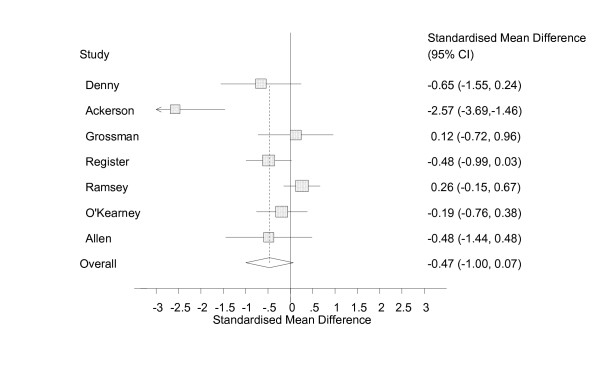
Analysis of emotional symptoms, random effects.

The random effects models showed a small effect size [67] with confidence intervals that included zero (ES = -0.14, 95% CI = -0.72 to 0.43, n = 5, figure 3) for the effect of self help technology on attitudes towards self, a medium effect size with confidence intervals that included zero (ES = -0.49, 95% CI = -1.23 to 0.25, n = 3, figure 4) for social cognition, and a medium effect size with confidence intervals that included zero (ES = -0.47, 95% CI = -1.00 to 0.07, n = 7, figure 5) for emotional symptoms.

## Discussion

### Effectiveness of self help technology

The systematic review is a key methodology in evidence based practice and the gold standard for the assessment of the effectiveness of interventions [51]. Overall, this review reveals a number of interesting findings. Despite the high prevalence of depression among adolescents, only two studies that involved self help technology for symptoms of depression were identified [54,56]. The bulk of the studies (n = 6) involved anxiety symptoms and six concerned other outcomes theoretically or empirically related to depression. Despite the explosion in the use of the internet, nearly half the studies involved written bibliotherapy.

In this review, effect sizes were calculated across 3 outcome measures: attitude towards self, social cognition, and emotional symptoms. The analyses indicated medium effect sizes for social cognition and emotional symptoms and a small effect size on attitudes towards self. However, all three meta analyses were statistically non-significant, although the effects on emotional symptoms approached significance (ES = -0.47, 95% CI = -1.00 to 0.07).

Many of the studies did not use samples from clinical populations. It is possible that concerns about the appropriateness of self help in adolescents has meant that researchers have tended to pilot these interventions in groups with more minor or circumscribed problems. As noted in the introduction, self help treatments may be especially useful for adolescents with early symptoms of anxiety, depression or emotional distress that do not reach diagnostic thresholds [22]. Further research using clinical populations may be required if these treatments are going to have more general utility.

In those cases where interventions are ineffective, this may reflect the lack of theoretical basis to the intervention, or poor uptake amongst clients. However, the descriptions of the theoretical basis of the interventions and their uptake were very limited, which made it difficult to examine the relationships between these factors and outcomes.

### Methodological issues

An extensive search strategy was used, involving the searching of five electronic databases, hand-searching nine journals, checking the reference lists of identified studies, contact with authors and regular updating of searches. However, the search had a number of limitations. First, grey literature was not searched systematically, which increased the possibility of missing unpublished studies; empirical evidence indicates that studies with positive results are more likely to be published [68,68]. However, the issue of including unpublished studies in systematic reviews is still controversial [68]. Some researchers argue against their inclusion, as such studies may have serious limitations that have prevented their publication [69]. Egger found in one meta analysis of 60 studies that unpublished studies had lower methodological quality than published trials and that they did not report important quality criteria such as concealment of allocation or blinding [70]. The funnel plot is a test that is used to examine publication bias in systematic reviews, but was not applicable in this review, due to the small number of studies [51].

The inclusion of only English language studies is another limitation of this study. Excluding trials reported in languages other than English may introduce bias and reduce the precision of results [68,71]. However, Egger found that meta analyses based exclusively on English language studies produced estimates close to those without any language restriction [72].

A large number of studies were identified because the search focussed on sensitivity rather than specificity. The initial check of titles and abstracts were undertaken by the first author alone. It would have been preferable to have these checked by both authors to ensure reliability, but time and resource limitations meant that this was not possible.

### Comparisons with other studies

The effectiveness of self help technology for emotional symptoms in the present analysis is similar to the analysis of 8 studies in adults with depression and anxiety in primary care, which reported a mean effect size of 0.41 [73], and a more recent review of 12 randomized controlled studies for internet-based cognitive behaviour therapy programs for symptoms of depression and anxiety [29] which reported a mean effect size of 0.40. However, in general, the effect size estimates from the current study were lower than the values that were reported by other meta analyses of the effectiveness of self help technology in adults. For example, Gould meta analysed 40 studies of self help for a wide range of problems (including depression, fear, headache, sleep and behavioural problems) and reported an overall effect size of 0.76, with an effect size of 0.74 in depression [27]. Cuijpers reviewed seven studies in unipolar depression and reported an overall effect size of 0.82 [26], while Marrs, in his review of 70 studies of a wide range of problems such as anxiety, depression, weight loss, and smoking, and reported a mean effect size of 0.57 for depression and 0.95 for anxiety [25].

These higher effect sizes in adults may be due to the difference between adolescent and adult populations (in terms of motivation and compliance with treatment) or methodological issues, such as differences in inclusion criteria (many of these meta analyses covered a wide range of problems) or the quality of the studies included.

One meta analysis investigated the effectiveness of bibilotherapy for depression among patients in three age groups: adult, adolescents and elderly. Based on five adolescent studies, the review found an effect size of 1.32 (95% CI = 0.90 to 1.73) [74]. However, this discrepancy may reflect a number of differences, including the definition of bibliotherapy, the amount of therapist contact, the review methodology (the Gregory study included randomised trials and pre and post single treatment group studies) and inclusion criteria (the Gregory review was restricted to depression).

### Quality assessment

In this study, quality assessment was done using the QRS [47] by two researchers working independently. The overall mean quality score of the studies included was low (16.4 out of 42). Therefore the results of the review can only be considered preliminary until the completion of more rigorous studies.

In addition to the rating of the quality of the studies, generalising the results of the review must be done with caution as there are significant limits to the external validity of the study findings. Few studies reported the number of eligible participants who took part in the study. Thirteen out of 14 studies were published in the USA and one in Australia. There are many factors that may affect the use of self help technology in other countries: the degree to which these technologies are acceptable to adolescents; the structure of the health and education systems; skills training of professionals in providing these interventions; education and skills of adolescents; socio-cultural issues such as stigma; and the quality of care in control groups. Furthermore, the review used an inclusive age range for adolescents, and the acceptability and effectiveness of treatments for students aged 18–25 may be very different for adolescents aged between 12 and 17. As noted above, all studies reported volunteer participants and only two studies reported the inclusion of clinical participants [54,55]. The participants included in the review may differ from those found in routine clinical settings in the severity of their problems, their willingness to participate in research, their motivation and adherence to treatment. However, it is also possible that the nature of self help treatments means that they will generally be used only with a proportion of adolescents who are willing and able to use them. As noted above, a number of studies included groups with circumscribed mental health issues (such as test, spider and dating anxiety) and the results may not generalise to clinical samples with more complex problems.

### The implications for practice

The implementation of self help technology for adolescents with emotional problems would be premature until further high quality randomized controlled studies are conducted. The potential benefits of self help technology (increasing access, decreasing costs) should be weighed against the possible risk of implementing these technologies without strong evidence of effectiveness.

### Implications for research

The findings indicated weak evidence for the use of self help technology in the management of emotional problems in adolescents. There is a need for randomized trials to provide rigorous evidence of the effectiveness and cost effectiveness of self help technology for adolescents with emotional problems, compared to usual care in order to measure the effectiveness of these technologies in decreasing emotional symptoms. Also there is need for randomized trials to compare the effectiveness of different types of self help technology (e.g. bibliotherapy and information technology) to find out which is more effective and acceptable. Further randomized trials are needed to evaluate the effectiveness of self help technology in the long term. Most of the randomized studies identified in the current review report short follow up periods of less than 6 months. Finally none of the included studies investigate the effectiveness of self help technology in relation to age, gender or other characteristics of the participants. Further research is needed to investigate important moderators of treatment effect [75,76].

## Competing interests

The authors declare that they have no competing interests.

## Authors' contributions

MA wrote the protocol, conducted the searches, data extraction and quality assessment of studies, and wrote the article. PB assisted with data extraction and quality assessment, and assisted with the writing of the article. Both authors conducted the meta analysis, and read and approved the final manuscript.

## Supplementary Material

Additional file 1 Table 2. Click here for file
