# Supplementary material for: The effectiveness of self help technologies for emotional problems in adolescents: a systematic review
Source: Child Adolesc Psychiatry Ment Health. 2008 Jul 23;2:20. doi: 10.1186/1753-2000-2-20 (PMC2500003; doi:10.1186/1753-2000-2-20)
Supplement: Additional file 1 — Table 2. [file 1753-2000-2-20-S1.doc]

**Table 2 Characteristics of the interventions and comparators**

| **Study** | **Intervention**  **Comparator** | **Description of intervention**  **Description of comparator** |
| --- | --- | --- |
| Ackerson  [54] | Bibliotherapy and phone  Delayed bibliotherapy | Cognitive bibliotherapy for Depression was Feeling Good (Buns, 1980) that has a 6th-grade reading level. Participants were given 4 weeks to complete the treatment intervention. During the intervention phase, weekly telephone calls were made to participants by the experimenter to collect information on the number of pages read and the number of exercises completed in the provided workbook. Counselling was not provided during these brief contacts.  Receiving treatment after 1 month and no treatment during delay period but they were telephoned weekly during their 4 week waiting period. |
| Allen  [65] | Programmed study counselling text  Control group | Subjects were provided with a 165 page programmed study counselling text but received no information on relaxation training. Once a week the participants had the opportunity to call the therapists to discuss problems and success in using manual. No phone call lasted more than 12 minutes.  Subjects received letters a week after the initial interviewing had been conducted stating that due to scheduling difficulties, they would not be able to join group. No expectancies concerning the availability of future treatment were set up |
| Buglione [55] | Computer plus audiotape  Group plus audiotape | Computerized treatment (Coping with tests) presented systematic desensitization, including verbal descriptions of test anxiety producing scenes and audiotaped Jacobsonian relaxation, plus modules on concentration training and success rehearsal. Participants instructed to begin each session with desensitization, and divide remaining time between other modules. Maximum 10, 60 minute sessions over six weeks plus audiotape to practice at home.  Group systematic desensitisation and rational-emotive discussion with audiotaped ‘Jacobsonian’ relaxation instructions twice a day for one week before remaining five weeks of treatment, and encouraged to practice at home, average 4.2 per group. |
| Denny  [62] | Group therapy plus videotape  Control group | Each therapy session began with the relaxation procedure. Afterwards, the subjects viewed a 12 minute videotape depicting various models handling spiders. There were 4 tapes used in this treatment condition, one for each therapy session.  Subjects in this condition were administered the pretest and the post test measures and were not seen in the interim. They were offered treatment at the conclusion of the study |
| Grossman  [58] | Manual  Delayed treatment | Subjects were instructed to read the manual and to complete the written exercises contained within the manual within two days. The subjects were instructed by the manual to arrange four practice dates with 4 different people of the opposite sex, one date per week, within four weeks. Letters were sent at two and three week intervals and they were called once to remind them about the time and date of post test session.  Offered the manual at the end of treatment. |
| Lenkowsky  [60] | Bibliotherapy  Books and meeting | The first bibliotherapeutic intervention group attended the 3 weekly sessions with literature relevant to the problems students faced, but no discussion group  3 book report and literature sessions with general interest books |
| O'Kearney  [56] | Internet based group  Control group | Self-paced interactive Internet program that aims to help people identify problems with depression, to help overcome these problems and to develop good coping skills. The program comprises 5 main modules, which each take 30–60 minutes to complete. Students are able to proceed through the program at their own pace. One researcher attended first session, teachers supervised subsequent sessions, but no details about amount of contact or type of contact. Each session was 45 minutes for 5 weeks.  Received usual personal development program scheduled by the school for the 5 weeks of the trial consisted of ad hoc discussion and physical activities. There was no specific discussion of depression during these activities. |
| Ramsey  [63] | Self help video, slide/tape, handouts, audiotapes  Control group | Five self contained stations completed without professional assistance, to be completed in one two hour and 30 minute session or two one hour and 15 minute sessions. First station was self assessment of response to stress, including 4 published scales. Station 2 involved 25 minute video containing information on stress, followed by 10 minute slide/tape presentation on cognitive restructuring. Third station involved second 21 minute videotape on effects. The fourth station involved relaxation audiotape and the fifth station involved stress control and management.  Subjects took all pre-test measures and were also assessed on all measures at the 30 day follow up. These subjects received no treatment. |
| Register  [57] | Manual without phone contact  No contact wait list control group | Manual including stress inoculation training procedures and content based on the three systems model of anxiety, and relaxation training based on the relaxation response, presentation of 5 coping strategies, distraction, imagining pleasant events, imagining sensations other than anxiety, and imagining anxiety in a different situation. No phone contact  No contact during the intervention and contacted 1 week after follow up and then therapists gave them the manuals. |
| Salt  [59] | Computer  Control group | A series of 10 guidance software programs which included teaching concepts of self awareness, goal setting, motivation, assertiveness and negotiating. These students were in the computer lab and received 12 sessions, 55 minutes, supervision by teacher but for technical issues only, daily feedback from students to earn co-operation point  Received no guidance and continued their regular course of instruction with their teacher and were administered only the pre and the post testing at the same time interval as were the experimental groups |
| Sandor  [37] | Bibliotherapy  Delayed treatment | 2 self help workbooks designed to provide a framework for problem solving, designed, tested and revised previously. Workbook consisted of 14 steps related to four basis steps of problems solving (problem definition, generation of alternatives, decision making, and verification). Completed in 30-60 minutes. Participants received one follow up call by researcher to answer any questions, ascertain whether additional books required, and reminder to continue to use the books.  Receiving workbook after 1 month. |
| Sheridan  [61] | Explicit bibliotherapy  Wait list control | 12 page handbook prepared by the senior author covered topics such as phases of personal reaction to loss of significant other, social problems, physical aggression, Santa Claus parents, dating, love, divorce, marriage, fears, and adolescents’ development. Also viewed a videotape on family matters. The participants read this book and did assignments. Group intervention consisted of three groups of 6, 5 and 5 students, each met for 45 minutes, five times, during 3.5 months.  Received standard individual counselling as needed, informed that the groups for which they had originally been volunteered would meet during the next school grading period because of limitations caused by staff-student ratios |
| Robinson  [66] | Computer based health education  Control group. | Health-Net includes four components: an electronic mail system, an electronic bulletin board, information and referral listings, and a self-help/ health information 'library'. The 'self help information' component includes information about over 90 selected health topics, including facts about pertinent resources and services available in the community. Over a 4 month intervention period, treatment group were mailed three additional newsletters encouraging Health-Net use  No intervention |
| Walker  [64] | Book and call phone  Control group | 9 page, 14 step workbooks for use by adolescents in dealing with hassles - upsetting situations in day to day living. These involved writing in a self identified hassle, completing a coping checklist, tallying coping responses, and reflecting on the balance of emotion and problem focused responses. Final section detailed alternatives to be put into action. Participants were contacted at least once during the 2 month intervention by telephone to monitor, not to supplement, the work intervention.  Received the intervention after the second post test |
